# Clinical significance of neutrophil-to-lymphocyte ratio as a predictor of lymph node metastasis in gastric cancer

**DOI:** 10.1186/s12885-019-6404-8

**Published:** 2019-12-05

**Authors:** Toshiyuki Kosuga, Tomoki Konishi, Takeshi Kubota, Katsutoshi Shoda, Hirotaka Konishi, Atsushi Shiozaki, Kazuma Okamoto, Hitoshi Fujiwara, Michihiro Kudou, Tomohiro Arita, Ryo Morimura, Yasutoshi Murayama, Yoshiaki Kuriu, Hisashi Ikoma, Masayoshi Nakanishi, Eigo Otsuji

**Affiliations:** Division of Digestive Surgery, Department of Surgery, Kyoto Prefectural University of Medicine, 465 Kajii-cho, Kamigyo-ku, Kyoto, 602-8566 Japan

**Keywords:** Gastric cancer, Gastrectomy, Neutrophil-to-lymphocyte ratio, Lymph node metastasis, Staging

## Abstract

**Background:**

Precise staging is indispensable to select the appropriate treatment strategy for gastric cancer (GC); however, the diagnostic accuracy of conventional modalities needs to be improved. This study investigated the clinical significance of the preoperative neutrophil-to-lymphocyte ratio (NLR) for the prediction of pathological lymph node metastasis (pN+) in GC.

**Methods:**

This was a retrospective study of 429 patients with GC who underwent curative gastrectomy. The predictive ability of NLR for pN+ was examined in comparison with that of computed tomography.

**Results:**

The preoperative NLR ranged from 0.6 to 10.8 (median, 2.0), and the optimal cut-off value for predicting pN+ was 1.6 according to the receiver operating characteristic curve with the maximal Youden index. Multivariate analysis identified a NLR ≥ 1.6 (odds ratio (OR) 3.171; 95% confidence interval (CI) 1.448–7.235, *p* = 0.004) and cN+ (OR 2.426; 95% CI 1.221–4.958, *p* = 0.011) to be independent factors associated with pN+ in advanced GC (cT2-T4). On the other hand, a NLR ≥ 1.6 was not useful for predicting pN+ in early GC (cT1). In advanced GC, a NLR ≥ 1.6 detected pN+ with a higher sensitivity (84.9%) and negative predictive value (NPV) (63.9%) than conventional modalities (50.0 and 51.7%, respectively). When the subjects were limited to those with advanced GC with cN0, the sensitivity and NPV of a NLR ≥ 1.6 for pN+ increased further (90.7 and 81.0%, respectively).

**Conclusion:**

The preoperative NLR may be a useful complementary diagnostic tool for predicting pN+ in advanced GC because of its higher sensitivity and NPV than conventional modalities.

## Background

Gastric cancer (GC) is the fifth most common cancer and the third leading cause of cancer-related death worldwide [1]. Gastrectomy with prophylactic lymphadenectomy is the basic surgical concept for GC, and treatment outcomes have been improving with advances in surgical techniques [2, 3]. In patients with early GC (EGC), D1/D1+ or D2 is adopted for those with cN0 or cN+, respectively [4]. On the other hand, in patients with advanced GC (AGC), D2 has been uniformly performed regardless of cN because the incidence of pN+ is high but difficult to precisely predict [4]. Preoperative assessment of lymph node metastasis is generally performed by computed tomography (CT) [5]; however, the diagnostic accuracy for pN+ is not sufficient and should be improved using another diagnostic tool.

Cancer-related inflammation was previously confirmed as a key determinant of cancer progression, and systemic inflammation is associated with alterations in peripheral blood leukocytes that are reflected in the neutrophil-to-lymphocyte ratio (NLR) [6–8]. Therefore, NLR may be a complementary diagnostic tool for pN+. There are many reports demonstrating the prognostic impact of NLR in different cancers [9–11]; however, few studies have examined whether NLR is useful for predicting pN+ in comparison with conventional diagnostic modalities.

In the present study, the diagnostic accuracy of NLR for pN+ was compared with that of conventional modalities such as CT. The aim of this study was to explore the clinical significance of NLR as a predictor of pN+ in GC, and to examine whether NLR can improve the diagnostic accuracy for pN+ in combination with conventional modalities.

## Methods

### Patients

Between January 2008 and May 2013, 578 patients underwent surgical treatment for GC at the Division of Digestive Surgery of Kyoto Prefectural University of Medicine (KPUM). Among these patients, this study examined only those who underwent CT followed by curative gastrectomy (R0). The following exclusion criteria were applied to potential subjects of this study: active infection, chronic inflammatory or autoimmune diseases, chronic use of steroid and/or immunosuppressive agents, hematological disorders, lack of information on preoperative complete blood counts, distant metastasis of GC, and simultaneous malignancies other than GC. In addition, to exclude the potential effects of treatment factors on the diagnostic accuracy for pN+, patients who underwent non-curative surgery (R1/R2) and those undergoing neoadjuvant chemotherapy (NAC) were also excluded. In contrast to Western countries, NAC has not been the standard treatment for GC even with advanced stage in Japan [4]. In total, 429 patients were included in this retrospective study.

### Assessment of NLR

The preoperative cell blood count (CBC) and differential white blood cell count (WBC), including neutrophils and lymphocytes, were measured within one week before surgery. NLR was calculated as the absolute neutrophil count divided by the absolute lymphocyte count.

### Assessment of cT and cN

All patients received upper endoscopy, barium meal examination, and chest and abdominal CT prior to surgery. The clinical T stage (cT) was assessed using upper endoscopy, barium meal examination, and abdominal CT findings by gastroenterologists and radiologists. The clinical N stage (cN) was diagnosed using the chest and abdominal CT findings by at least two radiologists. CT was performed at KPUM or Oike Clinic (Kyoto, Japan), a consociated medical center, employing a multidetector CT with 64 or 320 layers. Contrast-enhanced CT (CECT) with iopamidol or iohexol was the recommended standard, but patients who had iodine allergy, active asthma, or poor thyroid, heart, liver or renal function underwent plain CT without the contrast agents. Lymph nodes exhibiting a minor axis of 8 mm or greater or a major axis of 10 mm or greater on CT were regarded as “cN+” according to previous studies [5, 12, 13]. Endoscopic ultrasound (EUS) was not used for the evaluation of cN.

### Assessment of pT and pN

All patients underwent R0 surgery consisting of gastrectomy and lymphadenectomy based on the Japanese GC treatment guidelines (JGCTG) [4]. All resected specimens were microscopically examined by at least two pathologists, and the pathological T and N stages (pT and pN) were evaluated based on the current Japanese classification of GC (JCGC) [14].

### Statistical analysis

To evaluate the discriminatory ability of the NLR for pN+, receiver operating characteristic (ROC) curves were generated and the area under the ROC curve (AUROC) was measured. The optimal cut-off value of NLR was determined by the Youden index (*J*) [15, 16]. *J* is defined as the maximum vertical distance between the ROC curve and the diagonal or chance line and is calculated as *J* = maximum {sensitivity + specificity − 1} [15, 16]. Chi-square tests and Wilcoxon rank sum tests were used to compare categorical and continuous variables, respectively, between the two groups. In analyses of related factors for pN+, the clinical variables with *p* < 0.05 in the univariate analysis were incorporated into the multivariate analysis to identify independent factors. All statistical analyses were performed using JMP 13 (SAS Institute, Cary, NC, USA), and *p* < 0.05 was set as the level of significance.

## Results

### Patient characteristics

The clinical and pathological characteristics of the patients are shown in Table 1. Patients were diagnosed with cT1 (*n* = 277) or cT2-T4 (*n* = 152), and pathologically diagnosed with pT1 (n = 277) or pT2-T4 (n = 152). Seventy-three patients (17.0%) were diagnosed with cN+ by conventional diagnostic modalities, and 116 patients (27.0%) were pathologically diagnosed with pN+. The incidence of pN+ in cT2-T4 was 56.6%, which was higher than that (10.8%) in cT1 (*p* < 0.001). The preoperative NLR ranged from 0.6 to 10.8 (median, 2.0). The NLR value for patients with cT2-T4 was 2.6 ± 1.5 (mean ± standard deviation (SD)), which was higher than that (2.2 ± 1.2) for those with cT1 (*p* = 0.037).

**Table 1:** Clinicopathological characteristics of the patients

	cT1-T4 (*n* = 429)	cT1 (*n* = 277)	cT2-T4 (*n* = 152)	*P* value
Clinical characteristics				
Sex, n (%)				0.341
Male	278 (64.8)	175 (63.2)	103 (67.8)	
Female	151 (35.2)	102 (36.8)	49 (32.2)	
Age (years)				0.016
Median (range)	67 (29–89)	66 (35–89)	69 (29–89)	
Mean ± SD	65.6 ± 11.4	64.7 ± 11.2	67.1 ± 11.7	
Tumor location, n (%)				< 0.001
Upper	108 (25.2)	67 (24.2)	41 (27.0)	
Middle	204 (47.6)	154 (55.6)	50 (32.9)	
Lower	117 (27.3)	56 (20.2)	61 (40.1)	
cT, n (%)				–
T1	277 (64.6)	277 (100)	–	
T2	116 (27.0)	–	116 (76.3)	
T3	34 (7.9)	–	34 (22.4)	
T4	2 (0.5)	–	2 (1.3)	
cN, n (%)				< 0.001
N0 (negative)	356 (83.0)	267 (96.4)	89 (58.6)	
N+ (positive)	73 (17.0)	10 (3.6)	63 (41.4)	
NLR				0.037
Median (range)	2.0 (0.6–10.8)	2.0 (0.6–10.8)	2.1 (0.6–10.5)	
Mean ± SD	2.3 ± 1.3	2.2 ± 1.2	2.6 ± 1.5	
Pathological characteristics				
pT, n (%)				< 0.001
T1	277 (64.6)	245 (88.4)	32 (21.1)	
T2	49 (11.4)	16 (5.8)	33 (21.7)	
T3	62 (14.5)	14 (5.1)	48 (31.6)	
T4	41 (9.6)	2 (0.7)	39 (25.6)	
pN, n (%)				< 0.001
N0 (negative)	313 (73.0)	247 (89.2)	66 (43.4)	
N+ (positive)	116 (27.0)	30 (10.8)	86 (56.6)	

*SD* standard deviation, *NLR* neutrophil-to-lymphocyte ratio

### NLR according to cN and pN

The NLR value for patients with cN+ or cN0 was 2.4 ± 1.3 or 2.3 ± 1.3 (mean ± SD), respectively (*p* = 0.921). The NLR value for patients with pN+ was 2.6 ± 1.5 (mean ± SD), which was higher than that (2.2 ± 1.2) for those with pN0 (*p* = 0.003).

### ROC curve analysis

**Fig. 1:**
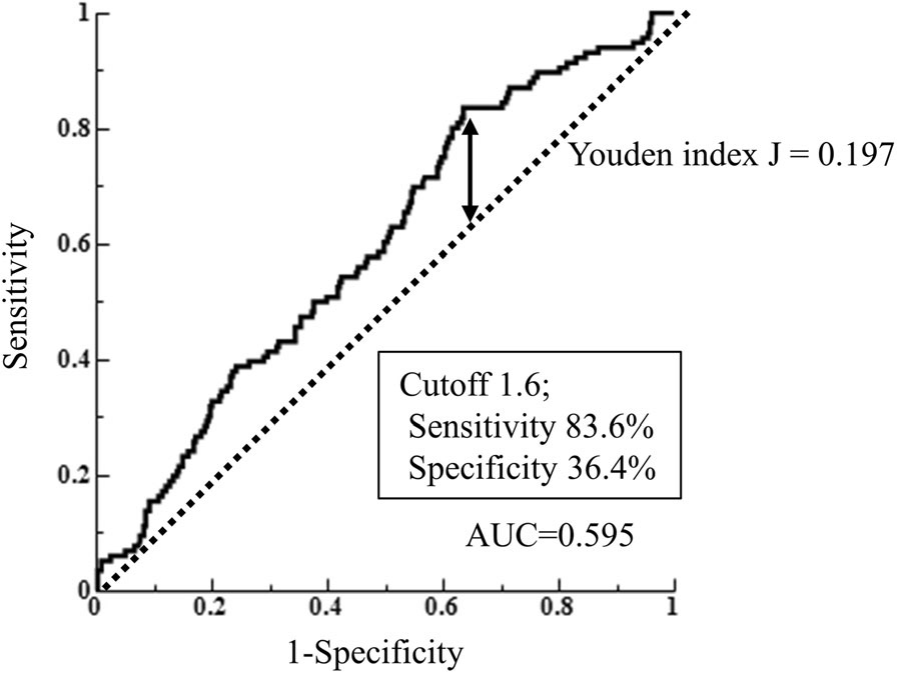
ROC curve for NLR for predicting pN+ in patients with gastric cancer. ROC: receiver operating characteristic, NLR: neutrophil-to-lymphocyte ratio, AUC: area under the curve

The optimal cut-off value of NLR for predicting pN+ was 1.6 according to the ROC curve using the maximal Youden index (AUROC, 0.595; sensitivity, 83.6%; specificity, 36.4%) (Fig. 1). Thereafter, all subjects were divided into two groups as follows: the low NLR (NLR < 1.6) and high NLR (NLR ≥ 1.6) groups.

### Clinical factors associated with pN+ in GC

The univariate and multivariate analyses of clinical factors associated with pN+ in patients with EGC (cT1) or AGC (cT2-T4) are shown in Table 2. In patients with cT1, the univariate logistic analysis showed that a NLR ≥ 1.6 was not significantly associated with pN+ (odds ratio (OR) 2.253; 95% confidence interval (CI) 0.942–6.266, *p* = 0.069), and cN+ was significantly correlated with pN+ (OR 9.680; 95% CI 2.537–37.07, *p* = 0.002). However, in patients with cT2-T4, the univariate and multivariate logistic regression analysis identified NLR (OR 3.171; 95% CI 1.448–7.235, *p* = 0.004) and cN (OR 2.426; 95% CI 1.221–4.958, *p* = 0.011) to be independently associated factors with pN+.

**Table 2:** Clinical factors associated with pN+ in patients with early or advanced gastric cancer

A: Early gastric cancer (cT1) (*n* = 277)						
Variables	Pathological lymph node metastasis (pN+) (*n* = 30)					
	Univariate			Multivariate		
	OR	95% CI	*p* value	OR	95% CI	*p* value
Sex			0.704			
Male	1					
Female	1.163	0.524–2.503				
Age			0.629			
< 65	1.205	0.561–2.590				
≥ 65	1					
Tumor location			0.353			
Upper	1.464	0.644–3.189				
Middle/Lower	1					
cN			0.002			
N0	1					
N+	9.680	2.537–37.07				
NLR			0.069			
Low (< 1.6)	1					
High (≥1.6)	2.253	0.942–6.266				
B: Advanced gastric cancer (cT2-T4) (*n* = 152)						
Variables	Pathological lymph node metastasis (pN+) (*n* = 86)					
	Univariate			Multivariate		
	OR	95% CI	*p* value	OR	95% CI	*p* value
Sex			0.424			
Male	1					
Female	1.325	0.666–2.679				
Age			0.496			
< 65	1					
≥ 65	1.263	0.644–2.476				
Tumor location			0.968			
Upper	1					
Middle/Lower	1.014	0.518–1.975				
cN			0.014			0.011
N0	1			1		
N+	2.300	1.183–4.573		2.426	1.221–4.958	
NLR			0.005			0.004
Low (< 1.6)	1			1		
High (≥1.6)	3.004	1.399–6.687		3.171	1.448–7.235	

*NLR* neutrophil-to-lymphocyte ratio

### Clinical value of NLR as a predictor of pN+ in advanced GC

The diagnostic accuracy of NLR or conventional modalities for pN+ in patients with AGC (cT2-T4) is shown in Table 3. The sensitivity, specificity, positive predictive value (PPV), negative predictive value (NPV), and diagnostic accuracy of a NLR ≥ 1.6 for pN+ were 84.9, 34.8, 62.9, 63.9, and 63.2%, respectively. Thus, the sensitivity was higher, specificity was lower, and diagnostic accuracy was slightly higher than those (50.0, 69.7, and 58.6%, respectively) of conventional modalities. Next, the predictive ability of NLR for pN+ was separately examined according to cN (N0 or N+) (Table 4). When the subjects were limited to AGC patients with cN0, the sensitivity and NPV of a NLR ≥ 1.6 for pN+ increased further (90.7 and 81.0%, respectively).

**Table 3:** Diagnostic accuracy for pN+ in patients with advanced gastric cancer

A: Conventional modalities (CT)			
	pN+	pN0	n
cN+	43	20	63
cN0	43	46	89
n	86	66	152
B: NLR			
	pN+	pN0	n
High NLR (≥1.6)	73	43	116
Low NLR (<1.6)	13	23	36
n	86	66	152
C: Diagnostic accuracy for pN+			
	cN+	High NLR (≥1.6)	
Sensitivity	50.0 % (95% CI: 43.2-56.2)	84.9 % (95% CI: 78.9-90.1)	
Specificity	69.7 % (95% CI: 60.8-77.8)	34.8 % (95% CI: 27.1-41.6)	
Positive predictive value	68.3 % (95% CI: 58.9-76.7)	62.9 % (95% CI: 58.5-66.8)	
Negative predictive value	51.7 % (95% CI: 45.1-57.7)	63.9 % (95% CI: 49.7-76.3)	
Diagnostic accuracy	58.6 % (95% CI: 50.8-65.6)	63.2 % (95% CI: 56.4-69.0)	

*NLR* neutrophil-to-lymphocyte ratio

**Table 4:** Predictive ability of NLR for pN+ separately examined according to cN in patients with advanced gastric cancer

A: cN0			
	pN+	pN0	n
High NLR (≥1.6)	39	29	68
Low NLR (<1.6)	4	17	21
n	43	46	89
Diagnostic accuracy for pN+			
	High NLR (≥1.6)		
Sensitivity	90.7 % (95% CI: 81.8-96.1)		
Specificity	37.0 % (95% CI: 28.6-42.0)		
Positive predictive value	57.4 % (95% CI: 51.7-60.8)		
Negative predictive value	81.0 % (95% CI: 62.7-92.0)		
B: cN+			
	pN+	pN0	n
High NLR (≥1.6)	34	14	48
Low NLR (<1.6)	9	6	15
n	43	20	63
Diagnostic accuracy for pN+			
	High NLR (≥1.6)		
Sensitivity	79.1 % (95% CI: 72.6-86.3)		
Specificity	30.0 % (95% CI: 16.0-45.5)		
Positive predictive value	70.8 % (95% CI: 65.0-77.3)		
Negative predictive value	40.0 % (95% CI: 21.3-60.7)		

*NLR* neutrophil-to-lymphocyte ratio

## Discussion

Systemic inflammatory response plays an important role in cancer development and progression [6–8]. Therefore, the increase in NLR, due to the systemic inflammatory response induced by cancer, may be a novel diagnostic modality for pN+ in GC. In the present study, the preoperative NLR was demonstrated to be independently associated with pN+ in patients with AGC (cT2-T4), but not in those with EGC (cT1). In AGC, a NLR ≥ 1.6 detected pN+ with a higher sensitivity (84.9%) than that (50.0%) of the CT. The high sensitivity of the preoperative NLR suggests it to be a useful complementary modality in the assessment of pN+ in AGC.

The therapeutic strategy for GC, including the extent of gastric resection and lymphadenectomy, is determined based on cT (T1 or T2-T4) and cN (N0 or N+) [4]. However, it is challenging to accurately predict the pathological tumor stage, particularly pN, because of the low diagnostic accuracy of conventional modalities [5, 12]. In the present study, the sensitivity, specificity, and diagnostic accuracy of CT for detecting pN+ (or pN0) in patients with AGC were as low as 50.0, 69.7, and 58.6%, respectively. On the other hand, the diagnostic accuracy for detecting pT2-T4 (or pT1) was 85.1% (Additional file 1: Table S1), which was easier to diagnose than pN. Positron emission tomography (PET) integrated with CT (PET-CT) may have played a role in the improved diagnostic accuracy for pN+ by increasing the specificity [5]; however, the low sensitivity is one of the weak points of this modality. Therefore, the development of novel diagnostic tools is essential to increase the specificity and sensitivity for predicting pN+.

Most previous studies focused on a high NLR as a useful predictor of long-term outcomes in patients with GC [10, 17, 18]. Indeed, in the targeted cohorts of the present study, the postoperative 5-year overall and cancer-specific survival rates of patients with a NLR ≥ 1.6 were significantly poorer than those with a NLR < 1.6 (data not shown). However, few studies have assessed the clinical significance of NLR as a diagnostic tool for pN+ in GC. Shimada et al. reported that the mean preoperative NLR in GC patients with pN+ was 2.91, which was significantly higher than that (2.40) in patients with pN0 [10], but the predictive ability of NLR for pN+ was not examined in detail. Zhang et al. estimated an optimal cutoff value of NLR of 2.0 (sensitivity, 52.6; specificity, 54.4; AUROC, 0.594), and a NLR ≥ 2.0 was significantly associated with pN+ by univariate analysis [18]; however, they neither assessed the influence of potential confounding factors, such as cT and cN, nor compared the diagnostic ability with conventional diagnostic modalities.

The present study was the first to explore whether the preoperative NLR is a predictor for pN+ independent of cN+, and to examine the diagnostic accuracy of NLR for pN+ in comparison with conventional modalities. As a result, a NLR ≥ 1.6 was found to be an independent predictor for pN+ in AGC (cT2-T4) with a slightly higher OR than conventional diagnostic modalities. In EGC (cT1), however, cN+ was not significantly correlated with pN+. Although the small sample size may be one of the responsible factors for the negative result in cT1, our study suggested that NLR was not superior to conventional modalities for predicting pN+ among patients with cT1. The correlation between the NLR value and cT may also have affected the results; however, the cut-off values of NLR for predicting pN+ determined by the ROC curve were 1.7 for cT1 and 1.6 for cT2-T4 (data not shown), which were both similar to the value for cT1-T4. Meanwhile, a NLR ≥ 1.6 may also be a good predictor of pT2/T3/T4 (vs pT1); however, the diagnostic accuracy (48.3%) was lower than that (85.1%) of conventional modalities such as upper endoscopy, barium meal examination and abdominal CT (Additional file 1: Table S1).

Although it has the potential to predict pN+ independent of cN+, the diagnostic accuracy of a NLR ≥ 1.6 for pN+ in AGC was only 63.2%, which was comparable to that of cN+. Actually, in the McNemar test, there was not a significant difference in the diagnostic accuracy for pN+ between a NLR ≥ 1.6 and cN+ (*p* = 0.175) (data not shown). As the low specificity was the major reason for the poor diagnostic accuracy, attention should be paid to the high incidence of false-positive cases when using NLR to predict pN+. On the other hand, the high sensitivity of NLR for pN+ was of note, i.e., a NLR < 1.6 may aid in the specific diagnosis of pN+. To clarify the most effective clinical use of NLR in combination with conventional diagnostic modalities, the predictive ability of NLR for pN+ was separately examined according to cN. When the subjects were limited to AGC patients with cN0, the sensitivity and NPV of a NLR ≥ 1.6 for pN+ increased further (90.7 and 81.0%, respectively). Therefore, a NLR < 1.6 may aid in the prediction of pN0, especially in combination with conventional diagnostic modalities.

The present study had several limitations that should be considered. First, the retrospective and single-center nature of the study may have generated selection bias in the cohort, and the number of study patients was relatively small, which may have limited the statistical power. Second, as the cut-off value of NLR was calculated only by a mathematical method, the low specificity of a NLR ≥ 1.6 for pN+ is a problem to be solved. Third, unfortunately, we could not find a correlation between preoperative NLR value and the number of pathological positive lymph nodes (Spearman’s rank correlation coefficient (ρ): 0.1224, *p* = 0.011) (data not shown); thus, it remains unclear whether biologically NLR is driving lymph node metastasis. Fourth, this study examined only NLR, but other immune-nutritional markers such as prognostic nutritional index (PNI) may also be useful predictors of pN+. However, to the best of our knowledge, this is the first report to present the novel potential of preoperative NLR for predicting pN in patients with AGC in comparison with conventional modalities. As the number of elderly patients with GC who have many comorbid diseases and poor organ function is increasing [19–22], limited lymph node dissection may be reasonable for such patients when pN0 is highly suspected based on the combined use of conventional modalities and NLR. The results of the present study and the optimal cut-off value of NLR need to be validated in further studies with large sample sizes to develop the sophisticated treatment strategies based on the reliable cN.

## Conclusion

The preoperative NLR may be a useful complementary diagnostic tool in the assessment of pN+ in AGC because of its higher sensitivity and NPV than conventional diagnostic modalities.

## Supplementary information

**Additional file 1: Table S1.** Diagnostic accuracy for pT2/T3/T4 in patients with gastric cancer.

## Data Availability

The datasets used and/or analyzed during the current study are available from the corresponding author on reasonable request.

## Abbreviations

AGC: Advanced gastric cancer
AUROC: Area under the receiver operating characteristic curve
CBC: Cell blood count
CECT: Contrast-enhanced computed tomography
CI: Confidence interval
CT: Computed tomography
EGC: Early gastric cancer
EUS: Endoscopic ultrasound
GC: Gastric cancer
JCGC: Japanese classification of gastric carcinoma
JGCTG: Japanese gastric cancer treatment guidelines
KPUM: Kyoto Prefectural University of Medicine
NAC: Neoadjuvant chemotherapy
NLR: Neutrophil-to-lymphocyte ratio
NPV: Negative predictive value
OR: Odds ratio
PET: Positron emission tomography
PET-CT: Positron emission tomography integrated with computed tomography
PNI: Prognostic nutritional index
PPV: Positive predictive value
ROC: Receiver operating characteristic
SD: Standard deviation
WBC: White blood cell count
