# Supplementary material for: Clinical significance of neutrophil-to-lymphocyte ratio as a predictor of lymph node metastasis in gastric cancer
Source: BMC Cancer. 2019 Dec 5;19:1187. doi: 10.1186/s12885-019-6404-8 (PMC6896257; doi:10.1186/s12885-019-6404-8)
Supplement: Supplementary file 1 — Additional file 1: Table S1. Diagnostic accuracy for pT2/T3/T4 in patients with gastric cancer. [file 12885_2019_6404_MOESM1_ESM.docx]

**Additional file 1: Table S1.** Diagnostic accuracy for pT2/T3/T4 in patients with gastric cancer

**A:** Conventional modalities (upper endoscopy, barium meal study and abdominal CT)

|  | pT2/T3/T4 | pT1 | n |
| --- | --- | --- | --- |
| cT2/T3/T4 | 120 | 32 | 152 |
| cT1 | 32 | 245 | 277 |
| n | 152 | 277 | 429 |

**B:** NLR

|  | pT2/T3/T4 | pT1 | n |
| --- | --- | --- | --- |
| High NLR (>1.6) | 114 | 184 | 298 |
| Low NLR (<1.6) | 38 | 93 | 131 |
| n | 152 | 277 | 429 |

**C:** Diagnostic accuracy for pT2/T3/T4

|  | pT2/T3/T4 | |
| --- | --- | --- |
|  | Conventional modalities | High NLR (>1.6) |
| Sensitivity | 78.9% (95%CI: 73.8-83.2) | 75.0% (95%CI: 69.1-80.4) |
| Specificity | 88.4% (95%CI: 85.6-90.8) | 33.6% (95%CI: 30.3-36.5) |
| Positive predictive value | 78.9% (95%CI: 73.8-83.2) | 38.3% (95%CI: 35.2-41.0) |
| Negative predictive value | 88.4% (95%CI: 85.6-90.8) | 71.0% (95%CI: 64.2-77.2) |
| Diagnostic accuracy | 85.1% (95%CI: 81.5-88.1) | 48.3% (95%CI: 44.1-52.1) |

NLR: neutrophil-to-lymphocyte ratio
